# Management of a First Branchial Cleft Cyst in a Pediatric Patient: A Case Report

**DOI:** 10.7759/cureus.108017

**Published:** 2026-04-30

**Authors:** Josiah Williams, Azwan Halim Abdul Wahab, Wan Ishlah Leman

**Affiliations:** 1 Department of Otolaryngology-Head and Neck Surgery, International Islamic University Malaysia, Kuantan, MYS

**Keywords:** branchial cleft cyst, facial nerve preservation, fistulogram, pediatric otolaryngology, work classification

## Abstract

First branchial cleft cysts are rare congenital anomalies in the pediatric population. The variable clinical presentations, ranging from asymptomatic neck masses to recurrent infections or otorrhea, pose significant diagnostic challenges, especially in young children, where anatomical landmarks are less defined, and misdiagnosis is common. This case report presents a detailed analysis of a Work type II first branchial cleft cyst in a one-year-old girl, emphasizing diagnostic strategies, imaging modalities, and individualized management considerations to optimize outcomes while minimizing complications such as facial nerve injury.

## Introduction

In pediatric patients, branchial cleft anomalies represent the second most common congenital neck masses, following thyroglossal duct cysts, as per a study by Handler et al. [1]. Various classifications exist for branchial cleft cysts; Work et al. classify the first branchial cleft cyst into two types [2]. Type I cysts are superficial to the facial nerve, with an external opening anterior and inferior to the tragus. Type II cysts exhibit a variable relationship with the facial nerve, typically opening at the posterior angle of the mandible. This case report describes the presentation, diagnosis, and management of a Work type II first branchial cleft cyst in a young child.

## Case presentation

A one-year-old girl with no comorbidities and an unremarkable postnatal history presented to the otolaryngology department with recurrent left neck swelling over the past six months. The swelling measured approximately 2 × 2 cm, with a punctum in the left submandibular region. Initially diagnosed as suppurative submandibular lymphadenitis, the patient underwent incision and drainage, along with gram-positive antibiotic coverage. Culture from the pus aspirate grew methicillin-sensitive *Staphylococcus aureus*.

Despite treatment, the swelling persisted. Subsequently, the child developed a new onset of otorrhea one month post incision and drainage. The nature of the otorrhea was a foul-smelling, persistent yellowish discharge with no associated hearing loss or dizziness. General examination was otherwise unremarkable, with intact facial nerve function. Otoscopic examination revealed pus discharge and keratin debris, obscuring the tympanic membrane.

An examination under anesthesia of the affected ear demonstrated dehiscence of the posterior external auditory canal wall, with keratin debris extending into the middle ear from the defect. The tympanic membrane showed a posteroinferior perforation, and ossicles were not visualized. Methylene blue instillation from the external sinus opening yielded no discharge in the ear canal.

**Figure 1:**
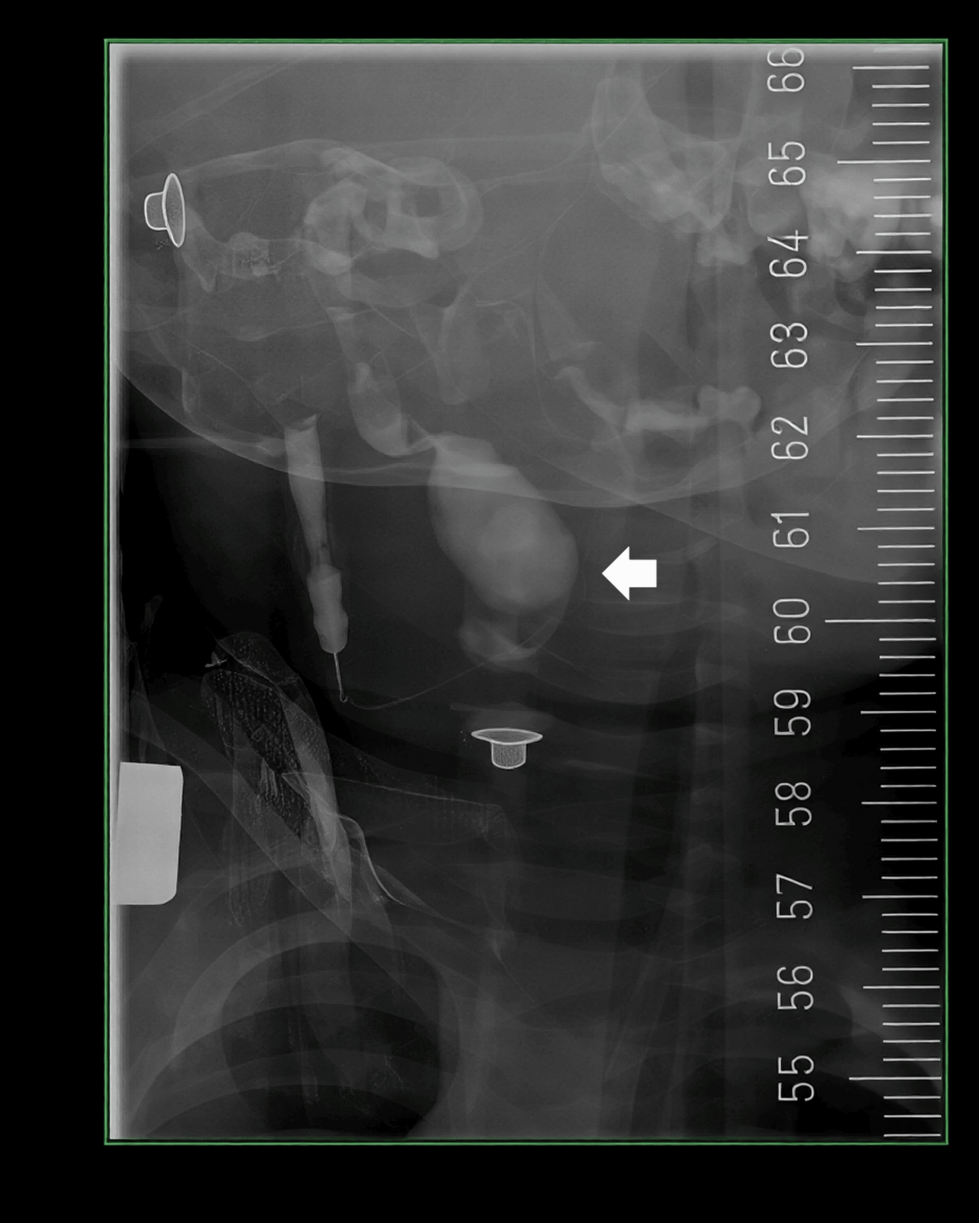
The fistulogram (anteroposterior view) illustrating the opacified tract from the submandibular punctum extending toward the ear (arrow pointing to opacified tract)

**Figure 2:**
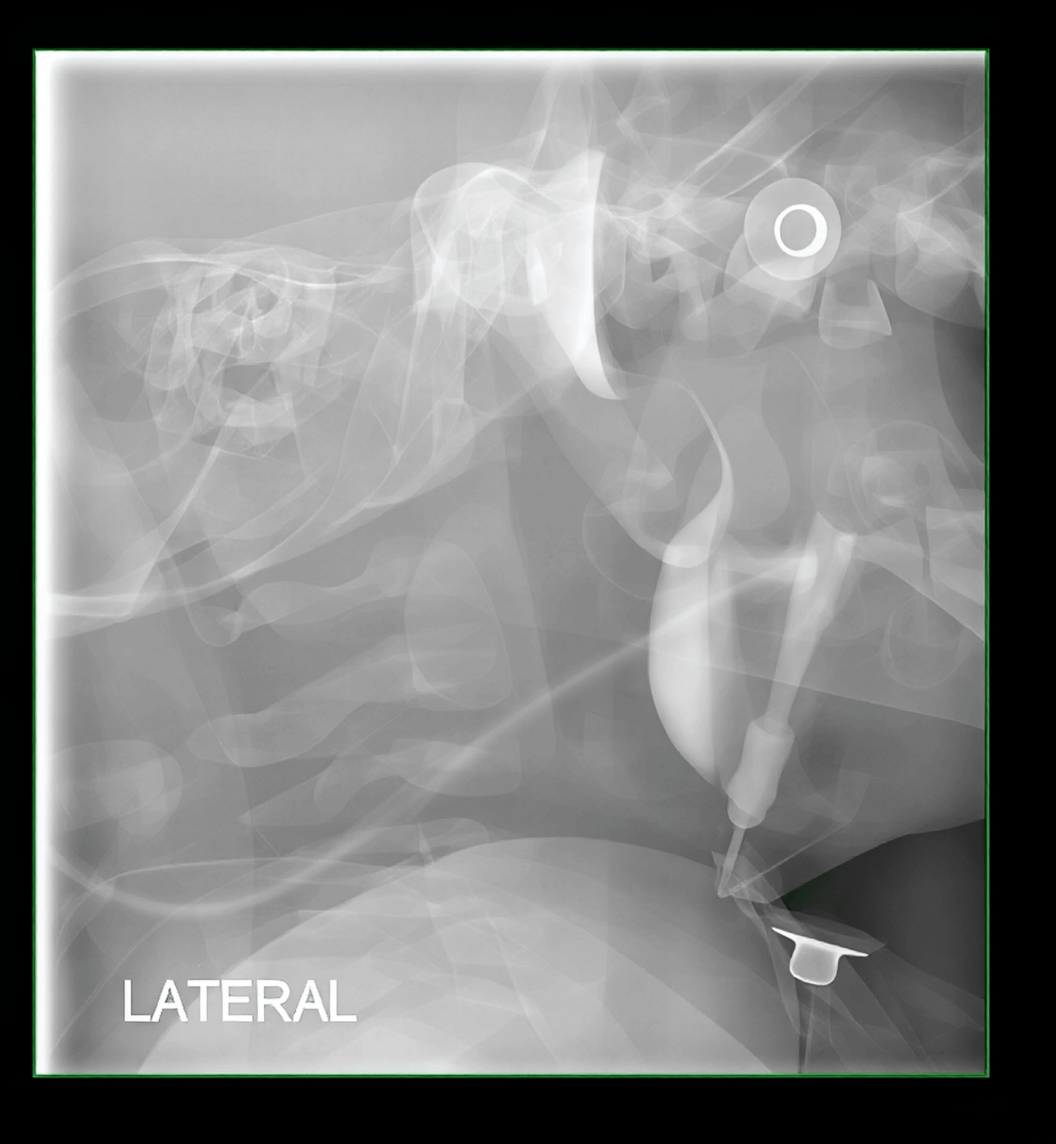
A fistulogram (lateral view) depicting the fistulous tract and associated collection in the neck

A fistulogram demonstrated an opacified tract extending superolaterally from the cutaneous opening toward the left ear, terminating in a collection in the left neck, indicative of a left first branchial cleft cyst with fistulous communication between the cutaneous opening and the external auditory canal (Work type II; Figures 1, 2).

Following multidisciplinary discussion with a pediatric otolaryngologist, conservative management with regular ear toileting and close monitoring with monthly follow-up was selected, given the tract's proximity to the facial nerve and the high risk of facial nerve palsy with surgery. Definitive surgical intervention was deferred until the child is older.

## Discussion

First branchial cleft anomalies account for a minority of branchial cleft malformations and often present diagnostic challenges due to their rarity and variable anatomy. In the pediatric population, these anomalies may manifest as recurrent neck swellings, otorrhea, or abscesses, as seen in this case. Initial misdiagnosis as lymphadenitis is common, leading to repeated interventions and increased recurrence risk.

The Work classification aids in surgical planning by delineating the cyst's relationship to the facial nerve as per Work et al. [2]. Type II cysts, as in this patient, pose greater surgical complexity due to their inconsistent association with the nerve. Imaging modalities, such as high-resolution computed tomography and fistulography, are essential for delineating the tract's course and its relation to critical structures, thereby guiding management and minimizing complications like facial nerve injury.

Studies emphasize the importance of superficial parotidectomy with facial nerve dissection for type II cysts, often performed at a mean age of six years to allow for better anatomical development and reduced surgical risk as per Triglia et al. [3]. Definitive treatment involves complete excision of the cyst and fistulous tract to prevent recurrence, which can be as high as 14%-22% following prior infection or incomplete resection, as shown in a study by Bajaj et al. [4]. Intraoperative facial nerve monitoring is recommended to preserve function. Techniques such as lacrimal probe insertion or dye instillation (e.g., methylene blue or gentian violet) can assist in tract identification during surgery, as illustrated by Quintanilla-Dieck et al. [5]. Non-essential structures, like segments of the cartilaginous external auditory canal, may be sacrificed if intimately involved with the cyst.

In this case, conservative management, which was regular ear toileting and monthly follow-up, was chosen due to the patient's young age and the tract's proximity to the facial nerve, aligning with recommendations to delay definitive surgery until acute infection resolves and the child matures, thereby optimizing outcomes and minimizing morbidity.

## Conclusions

Branchial cleft cysts are frequently misdiagnosed, leading to delayed definitive treatment and elevated recurrence rates. Optimal management entails complete excision of the cyst and tract while preserving the facial nerve. Comprehensive imaging is crucial to assess the anomaly’s relationship with surrounding structures. Treatment must be individualized, and in young children, deferred surgery may be appropriate to mitigate risks such as permanent facial nerve palsy.
